# Does the morphological fit between flowers and pollinators affect pollen deposition? An experimental test in a buzz‐pollinated species with anther dimorphism

**DOI:** 10.1002/ece3.2897

**Published:** 2017-03-19

**Authors:** Lislie Solís‐Montero, Mario Vallejo‐Marín

**Affiliations:** ^1^ Biological and Environmental SciencesSchool of Natural SciencesUniversity of StirlingStirlingUK; ^2^ Present address: Lislie Solís‐Montero, CONACYT. El Colegio de la Frontera Sur (ECOSUR) Unidad TapachulaTapachulaChiapasMéxico

**Keywords:** *Bombus terrestris*, herkogamy, pollen deposition, pollinator size, *Solanum rostratum*

## Abstract

Some pollination systems, such as buzz‐pollination, are associated with floral morphologies that require a close physical interaction between floral sexual organs and insect visitors. In these systems, a pollinator's size relative to the flower may be an important feature determining whether the visitor touches both male and female sexual organs and thus transfers pollen between plants efficiently. To date, few studies have addressed whether in fact the “fit” between flower and pollinator influences pollen transfer, particularly among buzz‐pollinated species. Here we use *Solanum rostratum,* a buzz‐pollinated plant with dimorphic anthers and mirror‐image flowers, to investigate whether the morphological fit between the pollinator's body and floral morphology influences pollen deposition. We hypothesized that when the size of the pollinator matches the separation between the sexual organs in a flower, more pollen should be transferred to the stigma than when the visitor is either too small or too big relative to the flower. To test this hypothesis, we exposed flowers of *S. rostratum* with varying levels of separation between sexual organs, to bumblebees (*Bombus terrestris*) of different sizes. We recorded the number of visits received, pollen deposition, and fruit and seed production. We found higher pollen deposition when bees were the same size or bigger than the separation between anther and stigma within a flower. We found a similar, but not statistically significant pattern for fruit set. In contrast, seed set was more likely to occur when the size of the flower exceeded the size of the bee, suggesting that other postpollination processes may be important in translating pollen receipt to seed set. Our results suggest that the fit between flower and pollinator significantly influences pollen deposition in this buzz‐pollinated species. We speculate that in buzz‐pollinated species where floral morphology and pollinators interact closely, variation in the visitor's size may determine whether it acts mainly as a pollinator or as a pollen thief (i.e., removing pollen rewards but contributing little to pollen deposition and fertilization).

## 1 Introduction

In plants with hermaphroditic flowers, the relative position of male and female sexual organs within a flower can mediate patterns of pollen export and receipt (Barrett, 2002b). Herkogamy, the spatial separation of the sites of pollen presentation and pollen receipt, has traditionally been interpreted as a mechanism to reduce self‐pollination (Richards, 1997), but it can also avoid physical interference between sexual functions and influence pollen transfer (Armbruster, Corbet, Vey, Shu‐Juan, & Shuang‐Quan, 2013; Barrett, 2002a; Fetscher, 2001; Webb & Lloyd, 1986). When the sexual organs are spatially separated, visitors can contact one set of sexual organs (male or female) at a time during a given visit, or they can touch both sexual organs but in different parts of the pollinator's body. If pollen placement and pollen pickup occurs in different parts of the pollinator body, pollen transfer can become less efficient (Armbruster et al., 2013; Webb & Lloyd, 1986). The problem of inefficient pollen placement in herkogamous flowers can be solved in different ways, including movement herkogamy (combination of sequential anther dehiscence and stamen repositioning; Armbruster et al., 2013), or possessing different floral morphs in which anthers and stigma are located in reciprocal positions (e.g., heterostyly and enantiostyly; Barrett, 2002b; Jesson & Barrett, 2002, 2003; Webb & Lloyd, 1986).

Enantiostyly is a type of reciprocal placement of sexual organs among flowers (Jesson & Barrett, 2003). Enantiostyly is characterized by the deflection of the style to either the left‐ or right‐hand side of the floral axis, with the anthers usually, but not always, placed opposite to the style resulting in mirror‐image floral morphs (Jesson & Barrett, 2002, 2003; Webb & Lloyd, 1986). Therefore, in enantiostylous species, pollen is deposited and picked up in opposite sides of the pollinator's body, and pollination occurs as visitors move between flowers of different morphs (Jesson & Barrett, 2005).

Across flowering plants, enantiostyly is often associated with heteranthery, the presence of two morphologically distinct types of anthers in the same flower (Jesson & Barrett, 2003). The two anther types represent the functional specialization of stamens into either pollination or feeding (Müller, 1883), as heterantherous species often use pollen as the main or only reward to attract pollinators (Vallejo‐Marín, Da Silva, Sargent, & Barrett, 2010). In species that combine enantiostyly and heteranthery, the reciprocal position of male and female sexual organs often involve the “pollinating” anthers but not necessarily the “feeding” anthers (Vallejo‐Marín et al., 2010).

In order for pollen to be reliably placed in and collected from specific locations in the pollinator's body, it is probably necessary for visitors to interact with the flower in a relatively predictable manner. Many heterantherous species have anthers that dehisce through small apical pores on the tips of the anthers (poricidal anthers) and are buzz‐pollinated (Vallejo‐Marín et al., 2010). Buzz‐pollination requires visitors, usually bees, to release pollen from poricidal anthers through the vibration of their thoracic muscles (Buchmann, 1983; De Luca & Vallejo‐Marín, 2013). When a pollinator approaches enantiostylous and heterantherous flowers, it grasps the feeding anthers, and vibrates to extract the pollen, which is ejected from the anther pores onto the ventral side of the pollinator's body (Bowers, 1975; Vallejo‐Marín, Manson, Thomson, & Barrett, 2009). During this process, the pollinating anther deposits its pollen on the side of the pollinator's body, which will then be transferred to the stigma when the insect visits a flower of the opposite floral morph (Whalen, 1979). Species that present complex floral morphologies such as those combining enantiostyly, heteranthery, and buzz‐pollination are great examples of close physical interactions between floral sexual organs and insect visitors.

The dynamic of pollen transfer in species with spatially segregated sexual organs also depends on the physical characteristics of the pollinator. The size of pollinators influences whether a pollinator makes contact with the sexual organs during visitation (Armbruster, Keller, Matsuki, & Clausen, 1989). For instance, studies on the relationship between proboscis length and depth of the floral structures that contain the reward (e.g., nectar spurs, corolla tubes) have shown that size matching between flower and pollinator can determine the success of pollen transfer (pollen deposition and removal; Kuriya, Hattori, Nagano, & Itino, 2015; Stang, Klinkhamer, Waser, Stang, & Van der Meijden, 2009). The overall size of pollinator can also be important, as body size relative to the flower determines which floral visitor pollinates and its efficiency (Armbruster & Muchhala, 2009; Nagano et al., 2014). Pollinator‐mediated selection on floral traits (Kuriya et al., 2015; Nagano et al., 2014) may optimize the mechanical fit between the floral sexual organs and the pollinator's body (Cresswell, 1998; Kuriya et al., 2015). Most previous studies in this area have focused on species providing nectar, oils, or scents as rewards (Armbruster & Muchhala, 2009; Kuriya et al., 2015; Nagano et al., 2014; Stang et al., 2009), and only a handful of studies have investigated size matching between pollinators and floral traits in pollen‐only reward flowers (Bowers, 1975; Duncan, Nicotra, & Cunningham, 2004; Gao, Ren, Yang, & Li, 2006; Kawai & Kudo, 2009; Liu & Pemberton, 2009).

Most of these studies mainly focus in describing whether floral visitors of different size make contact with the floral sexual organs when foraging for pollen (Bowers, 1975; Duncan et al., 2004; Gao et al., 2006; Liu & Pemberton, 2009; Solis‐Montero, Vergara, & Vallejo‐Marín, 2015). In general, these studies show that large‐ and middle‐sized visitors are more likely to make contact with the sexual organs compared to small visitors, which rarely touch the stigma. Less is known about the extent to which different sizes of visitors vary in their pollen transfer efficiency due to the closeness of the fit between the visitor and the floral sexual organs. We suggest that the degree of size matching between the pollinator body size and the floral sexual organ separation (herkogamy) is an important component in the reproduction of buzz‐pollinated plants with complex morphologies. We hypothesize that there should be an optimum size of visitor for a given size of flower that maximizes pollen deposition.

In this study, we address the hypothesis that there is an optimum size of visitor for a given size of flower that maximizes pollen deposition in *Solanum rostratum* (Solanaceae), a pollen‐only reward flower that possesses a relatively complex floral morphology combining enantiosty, heteranthery, and buzz‐pollination. We conducted an experimental test to determine how reproductive success relates to pollinator‐flower size matching in *S. rostratum* visited by buzz‐pollinating bumblebees (*Bombus terrestris*). *S. rostratum* is a self‐compatible, bee‐pollinated, annual herb that is partially outcrossing (outcrossing rate: *t *=* *0.70 ± 0.03; Vallejo‐Marín, Solís‐Montero, Souto Vilaros, & Lee, 2013), which inhabits open and disturbed habitats (Bowers, 1975; Whalen, 1979). This species strongly depends on pollinators to reproduce (Solís‐Montero, Vergara, & Vallejo‐Marín, 2015). The flowers of *S. rostratum* are presented in a vertical cyme, and are oriented horizontally, that is with the main floral axis parallel to the ground (Ushimaru, Dohzono, Takami, & Hyodo, 2009). This species is distributed from central Mexico to the Great Plains in the U.S.A. (Whalen, 1979) and also occurs as an invasive species in Canada, Asia, Europe, and Australia (Whalen, 1979; Zhao, Solís‐Montero, Lou, & Vallejo‐Marín, 2013). Pollinator observations conducted in North America reveal that *S. rostratum* is mainly visited by bees of different sizes (Bowers, 1975; Harris & Kuchs, 1902; Jesson & Barrett, 2005; Linsley & Cazier, 1963). While larger bees usually make contact with the stigma, smaller bees are precluded from making contact (Bowers, 1975). In central Mexico, natural populations are visited by 15 species of bees that range from 1 to 10 mm in thorax width. Legitimate pollinators of this species are large‐sized bees (from 5 to 10 mm) that contact the sexual organs during visitation. In contrast, illegitimate pollinators are small‐ and medium‐sized bees (from 1 to 4 mm), which do not make contact with the sexual organs, and mainly act as pollen thieves (Solís‐Montero et al., 2015).

The main goal of this study was to determine how pollination efficiency varies in relation to the size matching between the pollinator and the plant's sexual organs. We addressed two specific questions: (1) Is more pollen deposited on stigmas when the difference between the size of the pollinator (i.e., the width of the part of the pollinator's body that comes into contact with the sexual organs, in this case abdomen width) and the separation of the floral sexual organs is at a minimum? (2) Is fruit and seed production greater when the pollinator size closely matches the separation of the sexual organs? We expected that pollinator which fit closely with the floral sexual organs will deposit more pollen to stigmas and, consequently, increase fruit and seed production.

## 2 Materials and Methods

### 2.1 Floral morphology in native populations

**Figure 1:**
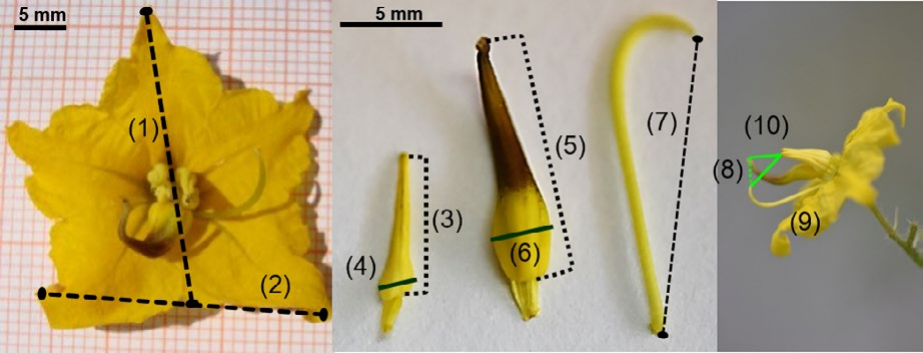
Ten floral traits measured in flowers of *Solanum rostratum*. (1) Corolla length and (2) width; (3) the length of one feeding anther and (4) the width of the base of this anther; (5) the length of a pollinating anther and (6) the width of the base of this anther; (7) the length of the style; the distances between: (8) the stigma and the pollinating anther, (9) the stigma and the nearest feeding anther and (10) the pollinating anther and the nearest feeding anther

In order to characterize the variation in floral morphology among natural populations of *S. rostratum*, we collected floral morphology data from six populations across a latitudinal gradient in Mexico during October and November of 2010 (Table 1). In each population, we measured between two and four flowers from 16 to 30 individuals (Table 1). For each flower, we measured the following ten traits with digital calipers: corolla length (1) and width (2); the length of the anther and the width of the anther at its widest point, for both the feeding (3, 4) and pollinating anther (5, 6); the length of the style (7); the distances between: the stigma and the pollinating anther (8), the stigma and the nearest feeding anther (9) and the pollinating anther and the nearest feeding anther (10; Figure 1). We analyzed these floral measurements using principal component analysis (PCA) of a correlation matrix. Differences among populations in the first two principal components were analyzed using an analysis of variances (ANOVA) of the principal component scores, and a Tukey post hoc test. For this analysis, we used JMP 7.0.2 (SAS Institute Inc. 2007) and plotted the results with Sigmaplot 13 (Systat Software Inc. 2015).

**Table 1:** Populations sampled for characterizing the floral morphology of *Solanum rostratum*

Pop. Code	Population	Latitude (N)	Longitude (W)	Elevation (m)	Number flowers measured (individuals)
AH	Atitalaquia, Hidalgo	20.07°	99.22°	2,090	60 (30)
CH	Cempoala, Hidalgo	19.91°	98.65°	2,467	32 (16)
PP	Puebla, Puebla	19.06°	98.16°	2,198	60 (30)
TEM	Teotihuacán, Estado de México	19.68°	98.86°	2,277	32 (16)
TP	Zapotitlán de Salinas, Puebla	18.33°	97.57°	1,670	120 (30)
VDU	Vicente Guerrero, Durango	23.74°	104°	1,926	60 (30)

### 2.2 Plant growth

In order to generate plants for the pollination experiment, we collected seeds from two of the six populations measured in the field (PP and VDU; Table 1). We selected these two populations because they exhibited the extreme values for the separation between the sexual organs within a flower (Figure 3). Seeds from 20 plants (hereafter maternal families) per population were extracted from the fruits and stored in paper bags at 5–7°C until planting. Five seeds per maternal family (5 × 20 = 100 plants per population) were planted in glasshouses at the University of Stirling with growth conditions as described in Vallejo‐Marín et al. (2013). After 4 weeks, plants were transplanted to 1.5‐L pots.

### 2.3 Pollination experiment

In order to investigate patterns of pollen deposition and both fruit and seed set, experimental plant arrays were exposed to visits by captive bumblebees (*Bombus terrestris* L). We chose this species of bumblebee for our experiment because individuals show considerable size variation (thorax width: 2.3–8.8 mm; Goulson, 2010), and colonies are readily available from commercial providers as they are used in the pollination of crops, including other buzz‐pollinated species such as tomatoes (*Solanum lycopersicum*). Moreover, bumblebees are native pollinators of *S. rostratum* in North America (Bowers, 1975), and *B. terrestris* has been previously used in pollination experiments with this species (De Luca & Vallejo‐Marín, 2013; De Luca et al., 2013).

Experimental arrays (35 blocks) consisting of 10 potted plants were placed in a flight cage (dimensions: 4 × 3 × 2 m) and exposed to visitation by captive bumblebees. Plants were arranged in two parallel rows of five plants, each placed 0.5 m apart and with 1 m of separation between each row. Each array contained five individuals from each of the two experimental populations (PP and VDU). We focused on the distance between the pollinating anther and the stigma because this should play an important role in pollen deposition due to the direct interaction between pollinator and this floral trait. During the pollination of *S. rostratum*, while a pollinator is collecting pollen from the feeding anthers, the pollinating anther touches one side of the pollinator's body and the stigma touches the corresponding position on the opposite side (Bowers, 1975).

The floral display of each plant in the array was standardized to four flowers (two for each enantiostylous morph); the remaining flowers were either removed or bagged with fine mesh to exclude bees. Each flower was individually labeled and the following floral traits measured: the distance between the stigma and the pollinating anther (8), the stigma and the nearest feeding anther (9), and the pollinating anther and the nearest feeding anther (10; Figure 1).

Each array (40 flowers from 10 plants per array) was exposed for 20 min to a single bumblebee, and the number of visits to each flower was recorded. A bee landing on a flower and contacting the sexual organs was scored as a visit. After 20 min, the bee was captured and the following five measurements were taken using digital callipers: the thorax width (1) and length (2), the abdomen width (3) and length (4), and the overall length of the bumblebee (5). To count the number of pollen grains deposited on the stigma, the terminal end of the style was collected from all the flowers of plants that received at least one visit. The top third of the style, including the diminutive stigma, was harvested after 24 hr and placed on a slide with fuchsine‐stained glycerol jelly (Kearns & Inouye, 1993). The 24‐hr delay between pollination and style collection was carried out to allow pollen tubes to grow and reach the ovary, as we were also interested in recording fruit and seed set in the experimental flowers. The total number of pollen grains deposited on each stigma was counted at 400 × magnification under a light microscope (Dialux 20EB, Leitz, Wetzlar, Germany). Six weeks later, we recorded whether fruits had formed and counted the number of seeds produced.

### 2.4 Size‐matching index (SMI)

**Figure 2:**
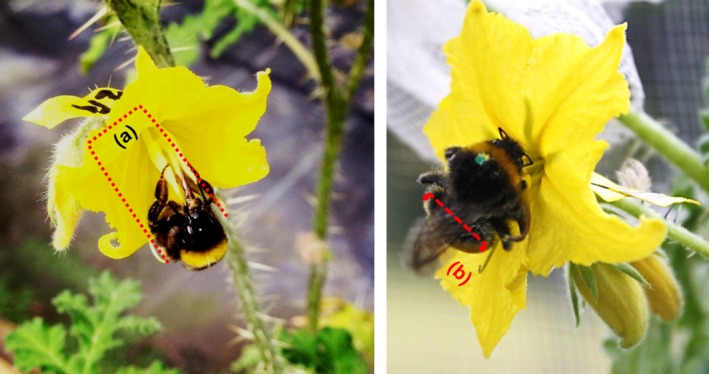
The size‐matching index (SMI) was defined as the difference between the distance from the pollinating anther to the stigma (a) and the bumblebee's abdomen width (b). Photograph by L. Bernstein and L Solís

We predicted that the degree of size‐matching between the spatial separation of the floral sexual organs and the body size of the visiting bumblebee would influence the probability of it contacting the anthers and stigmas and thus affect the number of pollen grains transferred between flowers. To investigate this hypothesis, we calculated the difference between the distance from the pollinating anther to stigma (DPAST), and the bumblebee's abdomen width (BAW) as shown in Figure 2. Hereafter we refer to this index as the size‐matching index or SMI (SMI = DPAST − BAW). The size‐matching index has a straightforward interpretation: when SMI = 0 the abdomen of the bumblebee fits exactly into the space between the pollinating anther and stigma. Positive values of SMI indicate that the space between the sexual organs is larger than the size of the bumblebee's abdomen, and thus, the bee cannot simultaneously touch both pollinating anther and stigma. Finally, negative values of SMI indicate that the separation between sexual organs is smaller than the abdomen's width of the visiting bumblebee, allowing for simultaneous contact of the pollinating anther and stigma during a visit.

### 2.5 Pollen deposition as a function of the size‐matching index

The variation in the SMI of the plant‐bee combinations used in this experiment is shown in Appendix S2. The number of pollen grains deposited by the bumblebee onto stigmas and the production of fruits and seeds were analyzed using separate generalized linear mixed models (GLMM). For these analyses, we used the statistical package *R* ver. 3.2.3 (R Core Development Team 2015). Mixed models were fitted with *lmerTest* package (Zeileis & Hothorn, 2002). The mixed models were visualized using the *plotLMER.fnc* function of the *languageR* package (Baayen, 2008), and we used the *optimix* package to plotting a quadratic term (Nash & Varadhan, 2011). The models used number of visits and SMI as fixed effects (including both linear and quadratic coefficients), and plant identity, array, and block as random effects. The best model for each response variable was selected by backward elimination comparing the log‐likelihood and Akaike information criterion of the nested models. Random effects that were not significant were eliminated from the model. Pollen deposition was natural log‐transformed (*ln (y + 1)*), and the model was fitted with a Poisson error term. In the case of fruit set, which can take values of either zero or one, and seed production (seed number), the models were fitted with a binomial error distribution (logit link) and a Poisson error distribution (log link), respectively. The variance and covariance of the random effects were obtained using the *ranef* function (package *lme4*).

## 3 Results

### 3.1 Floral morphology of *Solanum rostratum* in native populations

**Figure 3:**
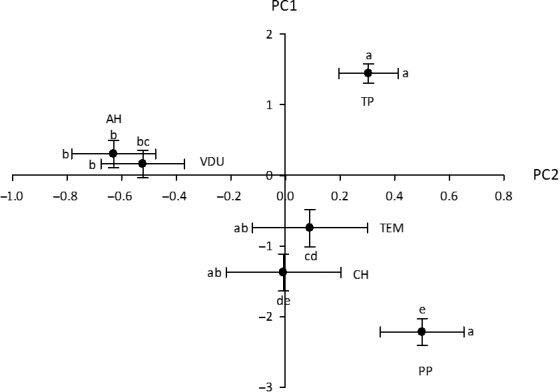
Mean values and standard errors of the principal component scores of the principal component analysis of floral traits. The mean scores for six native populations (AH, CH, PP, TEM, TP, and VDU) of *Solanum rostratum* are plotted, with principal component one (PC1) on the Y axis and principal component two (PC2) on the X axis. The lowercase letters indicate statistically significant differences among populations after a Tukey post hoc test. Population codes (uppercase letters) as in Table 1.

Throughout its distribution in Mexico, populations of *S. rostratum* differed in flower size, and in the separation between the sexual organs within its flowers. The first two components of the PCA on floral traits explained a total of 55% of the variance in floral morphology. The first principal component (PC1) explained 39% of this variance and was interpreted as reflecting flower size as almost all eigenvectors were positive and of similar magnitude (Table 2). Population PP had the smallest flowers (smallest PC1 values) and population TP had the largest (*F*_5,358_ = 56.86, *p *<* *0.0001; Figure 3). The second principal component (PC2) explained 16% of the variance and had the highest eigenvector scores for variables that defined the space separating the sexual organs (Figure 1 (8–10); Table 2). The southern populations (PP and TP) had more widely separated sexual organs than the northern populations (*F*_5,358_ = 9.42, *p *< 0.0001; Figure 3).

**Table 2:** Eigenvectors of the first two principal components (PC1 and PC2) of the principal component analysis of floral morphology traits in *Solanum rostratum*

Floral traits	PC1	PC2
Corolla L	0.402	−0.158
Corolla W	0.396	−0.142
FAnther L	0.368	−0.050
FAnther W	0.376	0.085
PAnther L	0.382	0.011
PAnther W	0.354	0.211
DPAST	0.125	0.631
ST	0.330	0.029
DFAPA	−0.020	0.384
DFAST	−0.090	0.595

L, length; W, width; FAnther, feeding anther; PAnther, pollinating anther; DPAST, the distance between the stigma and the pollinating anther; ST, the length of the style; DFAPA, the distance between the pollinating and the closest feeding anther; DFAST, the distance between the stigma and the closest feeding anther.

Both populations that were selected for the pollination experiment (PP and VDU) differed in the distance between the pollinating anther and the stigma (*F*_1,58_ = 5.50, *p *= 0.02; Appendix S1a) in the field, but this difference was not statistically significant in the progeny grown in the glasshouse in Scotland (*F*_1,35_ = 0.28, *p* = 0.60; Appendix S1b). However, we found enough variation in the distance between the pollinating anther and the stigma in the progeny of both populations (1.31–17.94 mm) to conduct the pollination experiment.

### 3.2 Pollination efficiency as a function of the fit between pollinator and floral morphology

#### 3.2.1 Number of pollen grains deposited by bumblebees on the stigma

We found pollen deposited on the stigmas of unvisited flowers in experimental arrays; 66% of unvisited flowers contained from 1 to 37 pollen grains. Pollen deposition on unvisited flowers may have occurred cause by artificial vibration of the anthers when the plants were transported from the glasshouse to the flight cage, or perhaps by unaccounted visits by native pollinators when setting up the experimental arrays. An alternative explanation is that there is automatic pollen deposition within the flowers of *S. rostratum*, although in the field, *S. rostratum* does not produce fruits through autonomous fertilization (Solís‐Montero et al., 2015), suggesting that spontaneous pollen deposition contributes little to reproduction under field conditions.

**Figure 4:**
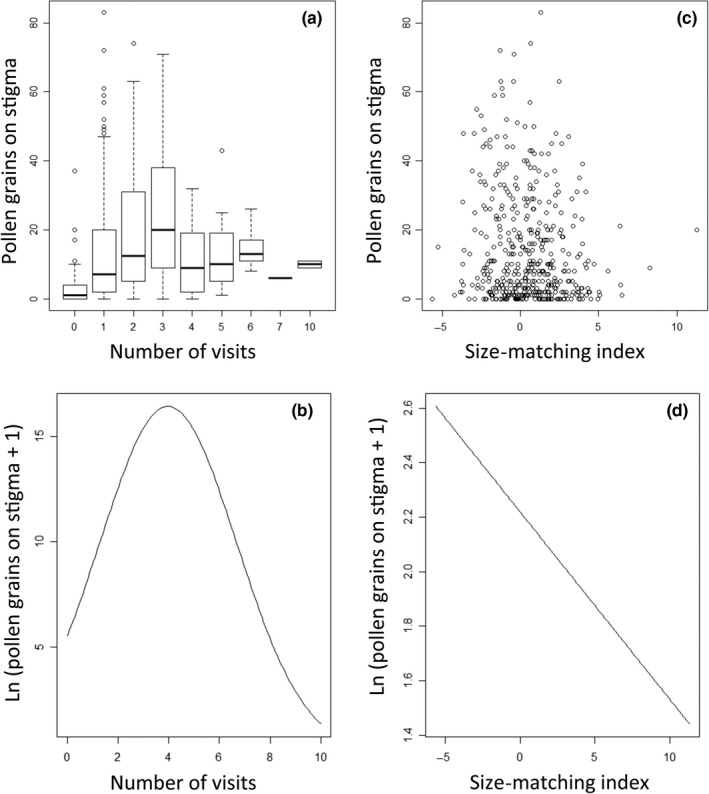
Pollen grains deposited on the stigma of *Solanum rostratum* as a function of (a) the number of visits conducted by *Bombus terrestris,* and (c) the size‐matching index, which measures the fit between the pollinator and the visited flower (positive values indicate that the distance between the floral sexual organs is larger than the width of the abdomen of the floral visitor). Lower panels show the fitted lines of the best‐fitting regression models of natural log‐transformed pollen grains on stigma [ln (pollen grains + 1)], as a function of (b) number of visits (quadratic model), and (d) the size‐matching index (linear model)

The number of pollen grains deposited on stigmas visited by pollinators had a quadratic relationship with visitation. Initially, more pollen grains are deposited with additional visits, but subsequently, pollen deposition decreases as visit number increases (Table 3; Figure 4a,b). In contrast to what we expected, pollen deposition on stigmas was linearly related to SMI (Figure 4c). We found a negative relationship between the number of pollen grains deposited on stigmas and the SMI (Table 3; Figure 4d). This means that when the abdominal width of a bumblebee is larger than the separation between the pollinating anther and stigma (negative values of SMI), more pollen grains are deposited on the stigma. Conversely, when the abdominal width of the bumblebee is smaller than this separation (positive values of SIM) fewer pollen grains are deposited onto the stigma.

**Table 3:** Summary statistics of the three generalized linear mixed models (GLMM). The values in parentheses are the standard error of the estimate for fixed effects and the standard deviation of the variance for random effects

Variable	Estimate (SE)	Test statistic (*z*)	*p* value
*Pollen grain deposition on stigmas*			
**Fixed effect**			
Number of visits	0.555 (0.030)	18.004	<0.001
Number of visits^2^	−0.069 (0.005)	−13.943	<0.001
Size‐matching index	−0.068 (0.008)	−7.858	<0.001
**Random effect**	**Variance (SD)**		
Individual per array	0.323 (0.569)		
Array‐block	0.273 (0.522)		
*Fruit production*			
**Fixed effect**			
Size‐matching index	−0.186 (0.111)	−1.675	0.094
**Random effect**	**Variance (SD)**		
Array‐block	0.799 (0.894)		
*Seed production*			
**Fixed effect**			
Size‐matching index	0.214 (0.037)	5.704	<.001
**Random effect**	**Variance (SD)**		
Individual per array	0.190 (0.436)		
Array‐block	0.082 (0.286)		

#### 3.2.2 Fruit and seed production in relation to the pollinator's fit with the floral sexual organs

**Figure 5:**
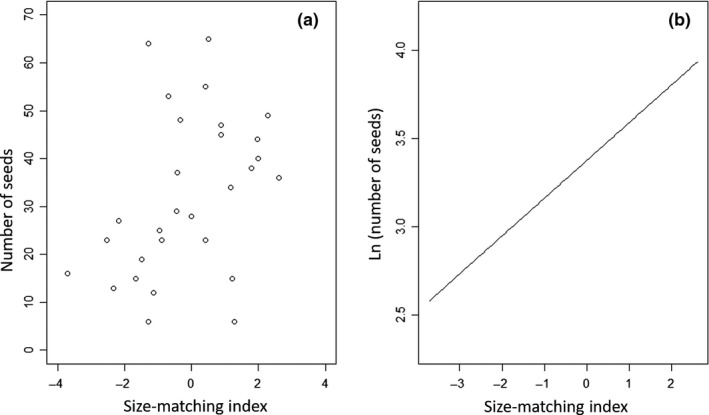
(a) Scatterplot showing the number of seeds produced by individuals fruits of the experimental plants against the size‐matching index between flowers of *Solanum rostratum* and *Bombus terrestris*. (b) Fitted line of the regression model on natural‐log transformed number of seeds [ln (number of seed)]

We found no significant effect of the SMI on fruit set (regression slope = −0.186; *p = *0.09; Table 3). In contrast, we found a positive relationship between the number of seeds and the SMI (Table 3; Figure 5). In other words, this intriguing result indicates that more seeds were produced when the bumblebee's abdomen was smaller than sexual organ separation than when the size of the bee's abdomen exceeded the distance separating anthers and stigma.

## 4 Discussion

Our survey of natural populations of *S. rostratum* indicates that populations of this species vary in floral size and in the degree of separation between sexual organs (anthers and stigma; herkogamy). Using experimental arrays and captive pollinators (*Bombus terrestris*), we have shown that the separation between sexual organs, relative to the size of the visiting pollinator, mediates patterns of pollen deposition on stigmas and seed set. Our results provide evidence in support of the hypothesis that correspondence in flower morphology and pollinator size is an important determinant of pollen transfer dynamics in buzz‐pollinated flowers with complex morphologies.

Native populations *S. rostratum* in Mexico varied in flower size as summarized by the first principal component (PC1). Populations with the largest flowers occurred in the north end of the native distribution, while the smallest flowers were observed in the south. The separation among flower organs (as measured by PC2) also showed variation among populations, but in this case, southern populations had the widest distance among anthers and stigma, while northern populations had the narrowest distance. The cause of this pattern of variation across a latitudinal gradient is unknown. One possibility is that variation in floral size and herkogamy may reflect in part exposure to different sizes of pollinators. For example, studies in nectar‐producing plants have shown that flower size can covary with the morphological characteristics of the local pollinator assemblage and that an optimal match between floral and pollinator size maximizes both male and female fitness components (Kuriya et al., 2015; Nagano et al., 2014). To the extent that populations of *S. rostratum* are exposed to assemblages of pollinators of different sizes, part of the floral variation we observed may be due to selection for an optimal match between flower and pollinator morphology.

The results from our experimental arrays revealed a quadratic relationship between pollinator visitation and pollen deposition. We found that initially, increased visitation resulted in more pollen grains deposited on stigmas, but that pollen deposition decreased after flowers received increasingly more visits. Previous studies have shown that the stigmas of other buzz‐pollinated plants are not saturated with pollen grains deposited during the first visit and continue receiving more pollen with additional visits (Kawai & Kudo, 2009). Studies in other species have also shown that more than one visit is required to achieve the maximum seed set (Snow & Roubik, 1987), although stigmas may become saturated with pollen after a few visits (>4; Kawai & Kudo, 2009). In *S. rostratum*, we found that the cumulative pollen deposition decreased after flowers received more than approximately three visits. A possible explanation for this is that when bumblebees visit the same flower many times in an experimental array they could remove pollen previously deposited on the stigma by direct contact of the pollinator body with the stigma or, indirectly, by vibrating the stigma when buzzing to obtain pollen (Dulberger, 1981). It is important to mention that in the experimental arrays the quantity of available pollen was finite, with only 40 flowers open at the same time. Thus, we speculate that at some point increased visitation may have removed pollen from stigmas at a higher rate than at which it was being deposited.

The variation in floral traits found in field populations of *S. rostratum* also provided us with the opportunity to test whether pollen deposition increases with the fit of the pollinator to the floral sexual organs. Pollination efficiency was estimated in our study through female fitness components, namely assessing the extent of pollen deposition onto the stigmas of the flowers as well as fruit and seed production. Instead of finding that pollen deposition was maximum when the flower and pollinator body matched best (near values of zero SMI) as we initially hypothesized, we found that pollen deposition in *S. rostratum* increased linearly with lower SMI values, i.e., when the visiting bee was larger than the separation of the sexual organs of the flower being visited. Consequently, pollen deposition was lowest when the abdomen of the bee (the part of the bee that may come into contact with anthers and stigmas) was smaller than the degree of herkogamy. A possible explanation for this result is that bees larger than the degree of herkogamy continue to contribute to pollen deposition as they are still able to touch the stigma (Armbruster et al., 1989). Conversely, when the bee is smaller than the degree of herkogamy, the visitor may touch the stigma more rarely and fewer pollen grains are deposited.

Contrary to our expectations, fruit set was not statistically associated with the size‐matching index (SMI). The regression coefficient of SMI on fruit set was negative (suggesting that bees larger than the separation of anthers and stigma are more likely to trigger fruit set than bees smaller than the degree of herkogamy), but not statistically significant (Table 3). This association is in the same direction as the one observed for pollen deposition, but further work using larger sample sizes is required to explore the effect of flower‐pollinator matching on fruit set. Alternatively, the lack of a statistically significant association between fruit set and SMI may instead reflect the fact that fruit production depends on other factors besides pollen receipt, such as the allocation of resources for sexual reproduction, growing conditions, and, in self‐compatible plants, the proportion of self‐ vs. outcross pollen (Montalvo, 1992; Obeso, 2004; Stephenson, 1981).

An intriguing, and unexpected, result of our study was that seed set (seed number per fruit) was positively related to SMI (Table 3). In other words, while visitation by bumblebees that were larger than the distance between the sexual organs, deposited more pollen grains (Figure 4), these visits resulted in fewer mature seeds per fruit (Figure 5). A potential explanation of these contradictory results is that when many pollen grains are deposited on the diminutive stigmas of *S. rostratum*, excess pollen causes stigma clogging and interferes with pollen tube growth. Another nonmutually exclusive explanation could be that higher rates of visitation increase the proportion of geitonogamous (self) pollen being deposited in the stigmas and that inbreeding depression causes the failure of self‐fertilized ovules. In our experiment, each individual plant in an experimental array had four flowers (two per floral morph) open at the same time. Therefore, pollinators could have transferred either self‐ or outcross pollen to the plant's stigma. The transfer of self‐pollen could occur between flowers of the opposite morph on the same plant (geitonogamy). In *Aquilegia caerulea*, for example, self‐pollination results in fewer seed being set because of a higher rate of seed abortion than with outcross‐pollination, which results from inbreeding depression during seed development (Montalvo, 1992). As the pollination experiment conducted in this study only registered the total amount of pollen deposited on the stigma and did not quantify the proportions of self‐ and cross‐pollen, further work would be needed to explore the fitness effects of self‐pollen saturation on *S. rostratum* stigmas.

Our study focused on pollen deposition and did not explicitly address how pollinator‐flower matching may affect pollen removal (male fitness). Although we did not measure pollen removal in our study, previous work on buzz‐pollinating bees, including bumblebees, suggests that pollinator size may affect the ability to remove pollen from flowers. For example, De Luca et al. (2013) found that heavier *Bombus terrestris* workers produced buzzes of greater amplitudes, which in turn resulted in larger amounts of pollen collected from flowers of *S. rostratum*. Thus, it is possible that our finding that larger bumblebees deposited more pollen grains, occurred not only because they matched or exceeded the distance between sexual organs, but also because they may have released and transported more pollen grains on their bodies. Further studies are needed to determine how pollinator‐size matching influences plant reproductive success via male fitness.

In general, our finding that the correspondence between bee size and the herkogamy mediates patterns of pollen deposition has implications for the functional role that visitors of different size play while visiting relatively complex flowers of buzz‐pollinated species such as *S. rostratum*. For example, visitor of a similar size or larger as the degree of herkogamy may functions as efficient pollinators, while smaller visitors on the same flowers may become functionally pollen thieves that remove pollen but fail to deposit it on the stigmas (Armbruster et al., 1989; Whalen, 1979). Our results show that size matching between the pollinator and the floral sexual organ separation determines the extent of pollen deposition in *S. rostratum* pollinated by captive bumblebees, but further work is required to determine whether the same phenomenon is observed in natural populations. We have shown that native populations of *S. rostratum* exhibit a large variation in the separation between the pollinating anther and stigma (from 3.45 to 14.25 mm). Furthermore, populations of *S. rostratum* in Mexico are visited by many bee species, which range widely in size (Solís‐Montero et al., 2015). Consistent with our experimental results, field observations indicate that small bees regularly fail to contact the sexual organs, and only mid‐ to large‐sized bees (from 4 to 20 mm) contact the stigma while collecting pollen (Solís‐Montero et al., 2015). Similarly, in invasive populations of *S. rostratum* in China, the effective pollinators of *S. rostratum* include large‐sized bees (e.g., *Xylocopa sinensis* and *Bombus ignites*; Zhang & Lou, 2015). Comparing the size matching between flower and visitors in different natural and invasive populations of *S. rostratum* would allow us to understand which bee species are likely to function as pollinators or as pollen thieves, at different geographic locations.

## 5 Conclusions

Due to the complex floral morphology (heteranthery and enantiostyly) of *S. rostratum*, and associated buzz‐pollination, it is crucial that pollinators fit closely with the sexual organs during the pollination process. Our results suggest that the size matching between a pollinator and the sexual organ separation determines the pattern of pollen deposition in *S. rostratum*. When the pollinator's body was wider than the separation of the sexual organs, more pollen grains were deposited on stigmas. However, we found that seed production not only depends on the quantity of pollen deposited but also may depend on other factors such as pollen competition and pollen quality (self‐ vs. outcross pollen). Understanding the relationship between flower‐pollinator matching and plant fitness will require integrating the effects of pollen removal and receipt, with postpollination processes, including pollen competition and the effect of inbreeding on seed maturation and survival. Nevertheless, we suggest that the physical matching between complex flowers and their floral visitor may be a useful predictor of whether a visitor is likely to behave as an effective vector for pollen transfer, or act as an inefficient pollinator or even become a pollen thief.

## Conflict of Interest

None declared.
