# Supplementary figures and images for: Does the morphological fit between flowers and pollinators affect pollen deposition? An experimental test in a buzz‐pollinated species with anther dimorphism
Source: Ecol Evol. 2017 Mar 19;7(8):2706–15. doi: 10.1002/ece3.2897 (PMC5395427; doi:10.1002/ece3.2897)

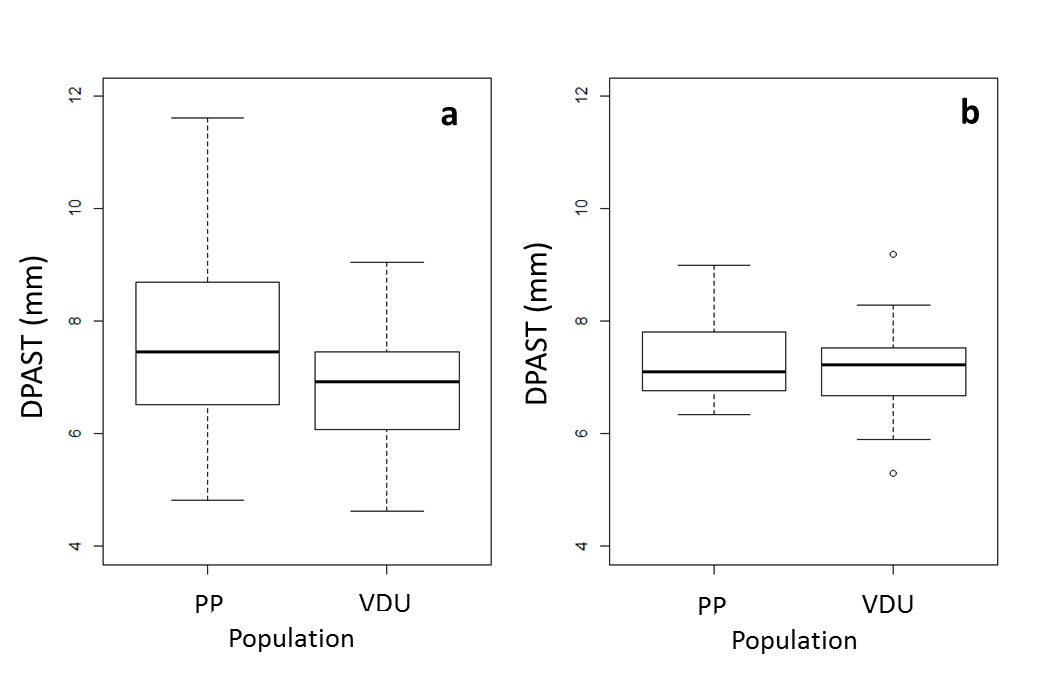

Supplement: Supplementary file 1 [file ECE3-7-2706-s001.tif]

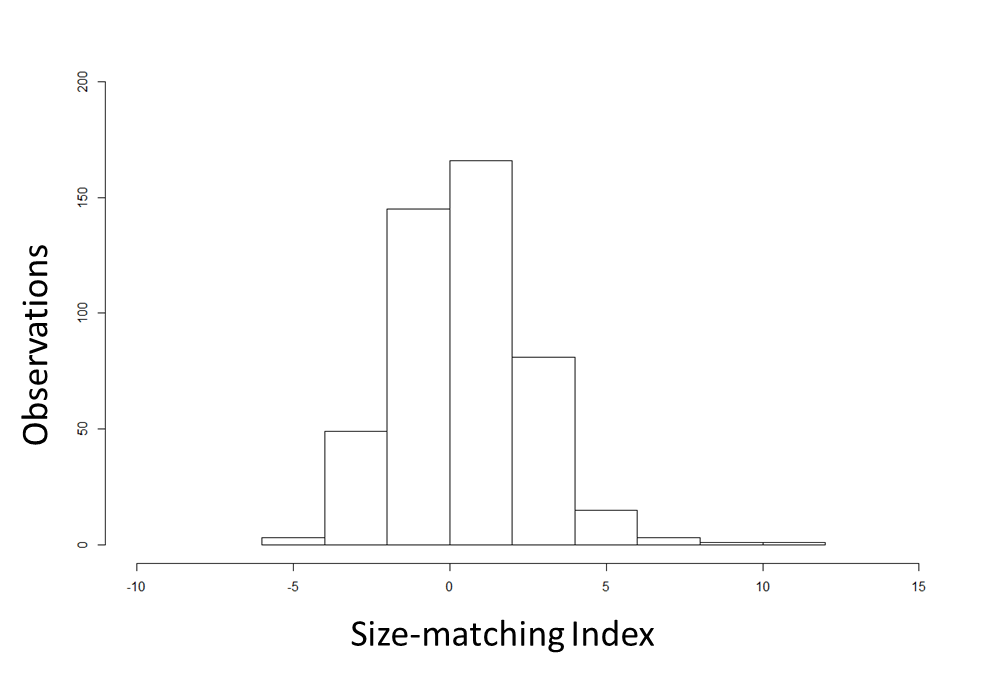

Supplement: Supplementary file 2 [file ECE3-7-2706-s002.tif]
